# Discriminatory Power Evaluation of Nuclear Ribosomal RNA Barcoding Sequences Through *Ophiocordyceps sinensis* Related Samples

**DOI:** 10.3389/fmicb.2018.02498

**Published:** 2018-10-23

**Authors:** Ping Zhang, Shenghui Cui, Xiu Ren, Shuai Kang, Feng Wei, Shuangcheng Ma, Bin Liu

**Affiliations:** ^1^National Institutes for Food and Drug Control, State Food and Drug Administration, Beijing, China; ^2^Department of Chinese Medicine Chemistry, Beijing University of Chinese Medicine, Beijing, China

**Keywords:** *Ophiocordyceps sinensis*, discriminatory analysis, Simpson index of diversity, nuclear ribosomal RNA barcoding sequences, ITS

## Abstract

Since the cost of *Ophiocordyceps sinensis* has increased dramatically and the counterfeits may have adverse effect to health, a rapid and precise species-level DNA barcoding identification system could be a potent approach and significantly enhance the regulatory capacity. The discrimination power of three subunits sequences from nuclear ribosomal RNA gene cluster were determined by Simpson’s index of discrimination using 43 wild *O. sinensis* fruiting bodies, pure cultures, commercial mycelium fermented powder and counterfeits. The internal transcribed spacer (ITS) sequences showed the highest variance and discrimination power among 43 samples, as determined by Simpson’s index of discrimination (*D* = 0.972), followed by large subunit (LSU; *D* = 0.963) and small subunit (SSU; *D* = 0.921). ITS-2 sequences showed the highest discrimination power for 43 samples among ITS-1, ITS-2, and 5.8S region of ITS sequences. All *O. sinensis* samples were grouped into a unique ITS sequence cluster under 95% similarity and two *O. sinensis* samples and six non-*O. sinensis* samples showed false claims. Our data showed that the ITS region could provide accurate species identification for *O. sinensis* samples, especially when macroscopic and microscopic method could not be applied in the highly processed commercial products. Since the authentication of *O. sinensis* related products is essential to ensure its safety and efficacy, identification of *O. sinensis* through ITS sequence comparison or unique PCR amplification of the species specific target, such as the ITS region, should be considered in the next revision of Chinese pharmacopeia.

## Introduction

*Ophiocordyceps sinensi*s, as a well-known Chinese caterpillar fungus, has been used in tonic and healthy food among Asia countries from the 15th century since it contains multiple valuable medicinal components determined by modern pharmacological science (Sun et al., 2017; Tsuk et al., 2017; Wang et al., 2017). *O. sinensis* is endemic to the Tibetan Plateau, including Tibet, Gansu, Qinghai, Sichuan, and Yunnan province (Yang et al., 2009). The cost of *O. sinensis* has increased dramatically because of the contradiction between limited natural resource and increasing demand. Its manufacture and sales were strictly regulated by the China Food and Drug Administration (CFDA) since 2016 because the natural fruiting bodies usually contain high amount of arsenic and other heavy metals^1^. Other *Ophiocordyceps* related fungi and the conidial form of the artificially cultured *O. sinensis* fermentation mycelia have also been used as substitutes in Chinese medicine and healthy food (Zhou et al., 2014; Cao et al., 2015).

Because non-*O. sinensis* species may have adverse effect to health, authentication of *O. sinensis* related products is essential to ensure its safety and efficacy. Traditionally, the identification of *O. sinensis* is through its morphological characteristics, but this method could not control the mixed final product authentication. In the last decade, fungi DNA barcoding has become a powerful tool to classify fungal species and provide an optimal option for the contradiction of traditional fungal classification criteria (Jensen et al., 1998; Zhong et al., 2010). Three subunits from nuclear ribosomal RNA gene cluster, including nuclear ribosomal internal transcribed spacer (ITS), large subunit (LSU), and small subunit (SSU) regions in *O. sinensis*, have been widely used in fungi identification (Xu, 2016) and the ITS region was formally recommended by the International Fungal Barcoding Consortium as the primary fungi barcode (Schoch et al., 2012). But for a specific genus, the discriminatory power of other barcodes might have higher resolving power for species discrimination, for example in lineages outside of Dikarya, ITS showed lower discriminatory power than nSSU and nLSU (Schoch et al., 2012). Identification of *O. sinensis* through DNA barcoding has been reported (Xiang et al., 2013), but limited data were available to characterize the discrimination power of different nuclear ribosomal DNA barcoding regions for *O. sinensis*.

In this study, the discrimination power of three subunits sequences from nuclear ribosomal RNA gene cluster were determined by Simpson’s index of discrimination using wild *O. sinensis* fruiting bodies, pure cultures, commercial mycelium fermented powder and counterfeits.

## Materials and Methods

### Sample Collection

From January 2015 to December 2016, a total of 40 *Ophiocordyceps* related samples were collected from Sichuan (*n* = 10), Qinghai (*n* = 8), Tibet (*n* = 7), Zhejiang (*n* = 5), Jiangxi (*n* = 4), Hubei (*n* = 2), Jiangsu (*n* = 2), and Yunnan (*n* = 2) provinces. An *Ophiocordyceps* reference material was obtained from the National Institutes of Food and Drug Control (NIFDC), and two reference strains were obtained from the China General Microbiological Culture Collection Center (CGMCC) (Table 1). The reference strains were stored in Brucella broth (BD, Beijing, China) with 50% glycerol at −80°C. Prior to testing, the reference strains were cultured on potato dextrose agar at 18 or 25°C until sufficient growth was obtained.

**Table 1:** 43 *Ophiocordyceps* related sample information included in this study.

Sample status	Claimed names^a^	No. of samples	Locations
Wild fruiting bodies	*Ophiocordyceps sinensis*	16	Qinghai (*n* = 6), Sichuan (*n* = 3), Tibet (*n* = 5), Yunnan (*n* = 1), Beijing (*n* = 1)^b^
	*Metacordyceps liangshanensis*	3	Sichuan (*n* = 3)
	*M. taii*	2	Qinghai (*n* = 2)
	*Cordyceps gunnii*	4	Sichuan (*n* = 4)
	*Black linen spine grass*	1	Tibet (*n* = 1)
	*O. gracilis*	1	Yunnan (*n* = 1)
	*Tibetan white grass*	1	Tibet (*n* = 1)
	Very grass^d^	2	HuBei (*n* = 2)
Strains	*Paecilomyces hepiali*	2	Jiangsu (*n* = 1), Beijing (*n* = 1)^c^
	*O. sinensis*	2	Jiangsu (*n* = 1), Beijing (*n* = 1)^c^
Fermented powder	*Ophiocordyceps* mycelium	4	Jiangxi (*n* = 4)
	*O. sinensis* mycelium	5	Zhejiang (*n* = 5)

*^a^Sample names when they were collected; ^b^Reference material from national institutes of food and drug control (NIFDC); ^c^Reference strain CGMCC3.7845 and CGMCC3.14243 from China General Microbiological Culture Collection Center (CGMCC); ^d^Commercial name of an O. sinensis product from the wild fruiting bodies.*

### PCR Amplification and Sequence Analysis

The total genomic DNA from 43 *Ophiocordyceps*-related samples, including 40 *Ophiocordyceps* related samples, two reference strains and one reference material, was extracted using a DNeasy plant mini kit (Qiagen, Germany), and the DNA concentrations were quantified using a Qubit fluorometer (Invitrogen, Shanghai, China). Three nuclear ribosomal gene regions, including the SSU, LSU, and ITS regions, were amplified by PCR, as described previously (Schoch et al., 2012). The following primers were used: LSU-F: ACCCGCTGAACTTAAGC, LSU-R: TCCTGAGGGAAACTTCG; SSU-F: GTAGTCATATGCTTGTCTC, SSU-R: CTTCCGTCAATTCCTTTAAG; ITS-F: TCCTCCGCTTATTGATATGC, ITS-R: GGAAGTAAAAGTCGTAACAAGG (Schoch et al., 2012). All PCR products were cloned into the pMD18-T plasmid (Takara Biotechnology Corp., Dalian, China) for sequence analysis at TianyiHuiyuan Biotechnology Corp. The obtained sequences were analyzed using the Sequencher 4.6 software (Gene Codes Corp., Ann Arbor, MI, United States). The search for homologous sequences was performed using the BLAST program at the US National Center for Biotechnology Information (NCBI) website^2^. The nucleotide sequences identified in this study were deposited into the GenBank.

### Comparison of the Discriminatory Power of Three Nuclear Ribosomal Genes

Multiple DNA sequence comparisons were performed with the BioNumerics 7.6 software (Applied Maths, Belgium), and the phylogenetic tree indicating relative genetic similarity was constructed on the basis of the neighbor-joining method. The discriminatory power of the *SSU*, *LSU*, and *ITS* sequence variations were compared, and Simpson’s index of diversity (D) was calculated using the following equation as described previously (Hunter and Gaston, 1988; Dillon et al., 1993):

$$D=1-\frac{1}{N(N-1)}\sum_{j=1}^{s}n_{j}(n_{j}-1)$$

where *N* is the total number of *Ophiocordyceps-*related samples in the study, *s* is the total number of types described by each method, and *n_j_* is the number of samples belonging to the *jth* type. DNA sequence types were defined as DNA sequences sharing 100% similarity and clusters were defined as ≥95% similarity (C1, C2, C3, …).

## Results

### Discriminatory Power Comparison of Three Nuclear Ribosomal Genes

**FIGURE 1:**
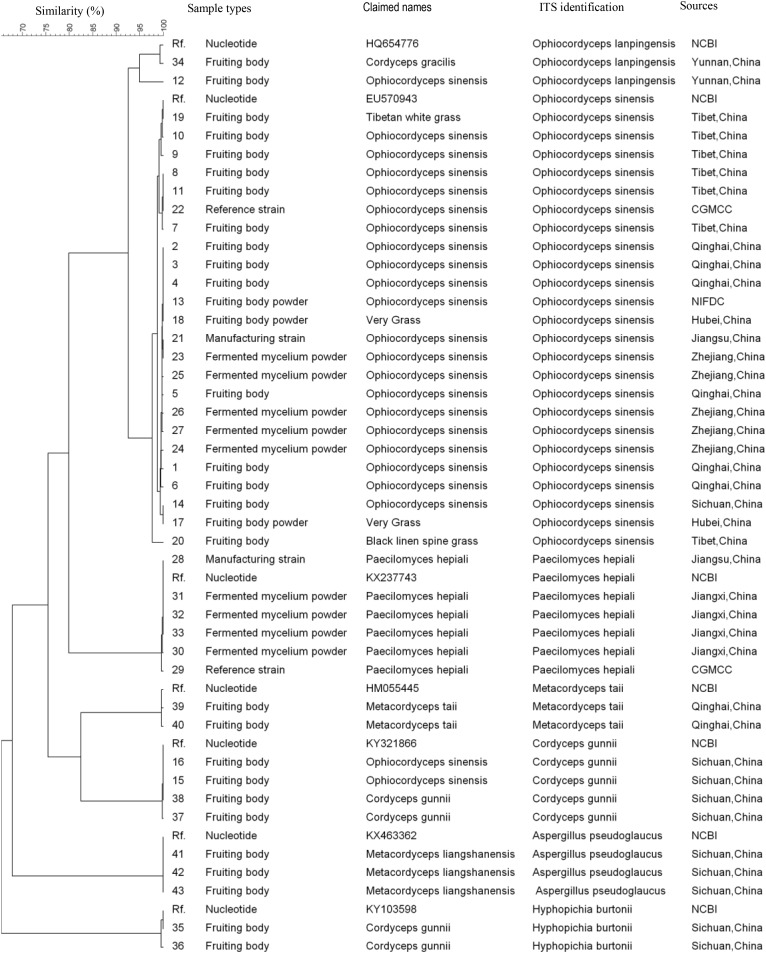
Discrimination of 43 *Ophiocordyceps* related samples through the internal transcribed spacer (*ITS*) sequences.

**FIGURE 2:**
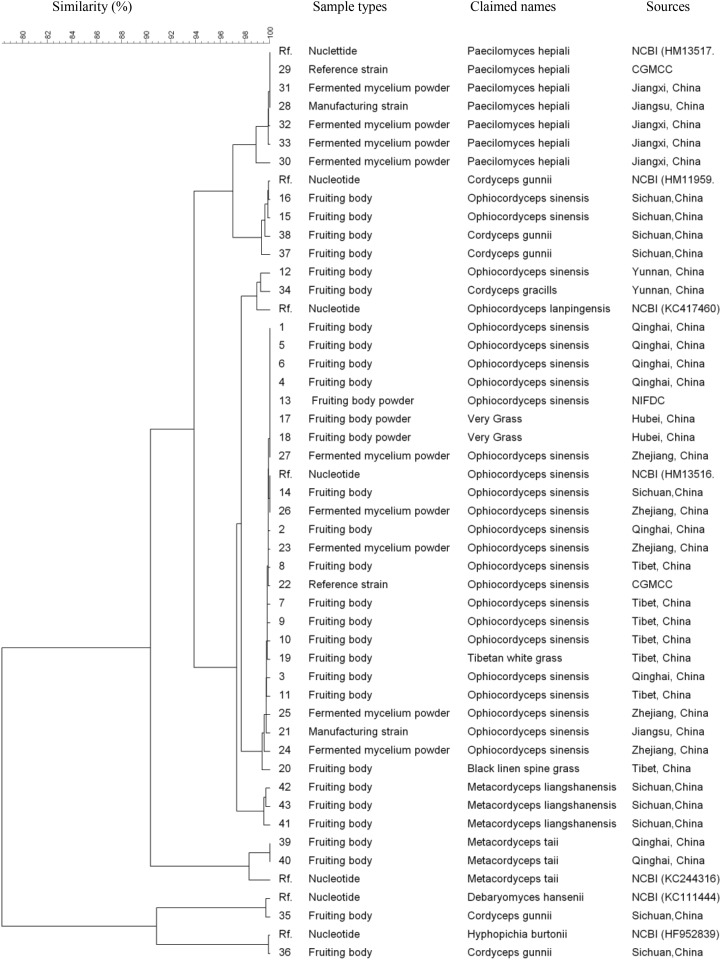
Discrimination of 43 *Ophiocordyceps* related samples through the large subunit (*LSU*) sequences.

**FIGURE 3:**
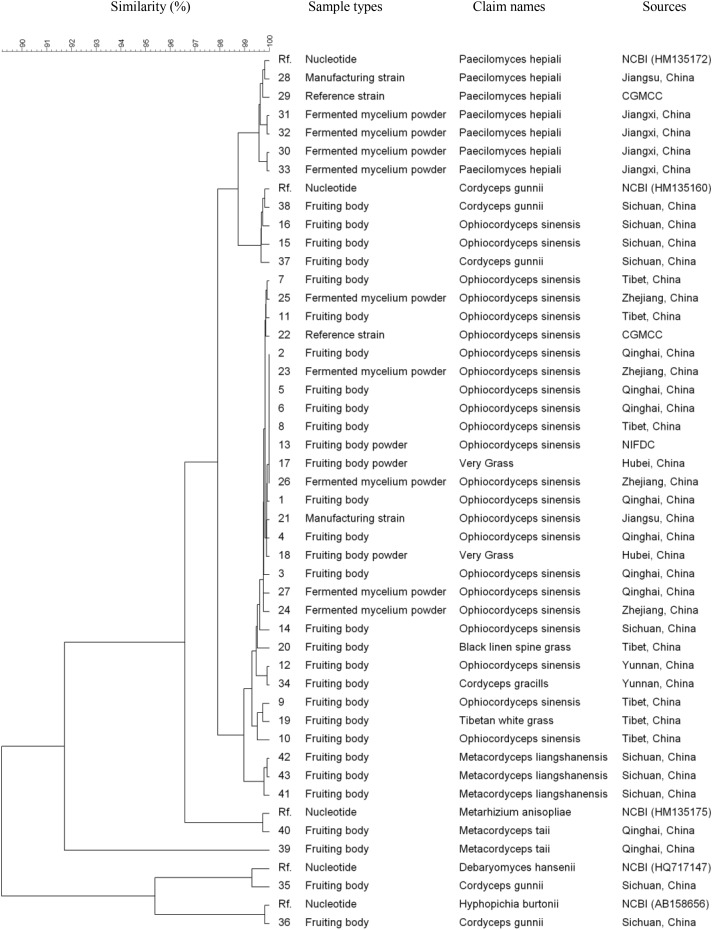
Discrimination of 43 *Ophiocordyceps* related samples through the small subunit (*SSU*) sequences.

All 43 samples were successfully amplified by ITS, LSU, and SSU universal primers. Overall, the ITS sequences showed the highest variance and discrimination power among 43 samples, as determined by Simpson’s index of discrimination (*D* = 0.972), followed by LSU (*D* = 0.963) and SSU (*D* = 0.921) (Table 2). The size of the largest ITS sequence type included five samples compared to eight samples of LSU and SSU sequences. Seven, four and three clusters were identified for ITS, LSU, and SSU sequences using 95% similarity as the cut-off value, respectively. The largest cluster of ITS, LSU, and SSU sequences included 24 samples of one species, 29 samples of two species and 40 samples of four species, respectively (Figure 1–3).

**Table 2:** Discriminatory power evaluation of three nuclear ribosomal RNA genes for 43 *Ophiocordyceps* related samples.

Gene regions		No. of types	Size (%) of the largest type	Discrimination index
SSU		36	8 (19%)	0.968
LSU		32	8 (19%)	0.963
ITS		28	5 (12%)	0.972
	ITS1	14	12 (28%)	0.884
	ITS2	24	7 (16%)	0.949
ITS	5.8S	14	19 (44%)	0.787
	ITS1 + ITS2	28	5 (12%)	0.968
	ITS1 + 5.8S	21	9 (21%)	0.930
	5.8S + ITS2	25	6 (14%)	0.956

Among ITS-1, ITS-2 and 5.8S region of ITS sequences, ITS-2 sequences showed the highest discrimination power for 43 samples, as determined by Simpson’s index of discrimination (*D* = 0.949), followed by ITS-1 (*D* = 0.884) and 5.8 S (*D* = 0.787).

### Species Identification Through ITS Sequences

Among 18 wild fruiting body samples claimed as *O. sinensis*, the ITS sequences of 15 samples showed 99–100% homology with the *O. sinensis* sequences deposited in the GenBank and two samples from Sichuan province showed 100% homology with the *Cordyceps gunnii* sequences deposited in the GenBank, one sample from Yunnan showed 100% homology with the *O. lanpingensis* sequences deposited in the GenBank. The ITS sequences of one Tibetan white grass sample and one Black linen spine grass sample showed 98–100% homology with the *O. sinensis* sequences deposited in the GenBank.

The ITS sequences of three *Metacordyceps liangshanensis* wild fruiting body samples, two *O. hawkesii* wild fruiting body samples and one *O. gracilis* wild fruiting body sample showed 95–100% homology with *Aspergillus pseudoglaucus*, *Hyphopichia burtonii* and *O. lanpingensis* sequences deposited in the GenBank, respectively. The ITS sequences of two *M. taii* wild fruiting body samples and two *O. gunnii* wild fruiting body samples showed 99% homology with the *M. taii* or *O. gunnii* sequences deposited in the GenBank, respectively. The ITS sequences of five *O. sinensis* mycelium fermented samples and two *O. sinensis* strains showed 99–100% homology with the *O. sinensis* sequence deposited in the GenBank. The ITS sequences of four *Ophiocordyceps* mycelium fermented samples and two *Paecilomyces hepiali* strains showed 99–100% homology with the *P. hepiali* sequence deposited in the GenBank.

## Discussion

In our study, the discrimination power of three subunits sequences from nuclear ribosomal RNA gene cluster were determined by Simpson’s index of discrimination using *O. sinensis* fruiting bodies, pure cultures, commercial mycelium fermented powder and counterfeits, the ITS region showed the highest discrimination power for 43 tested samples which was similar as shown in previous DNA barcode study for fungi (Schoch et al., 2012). All *O. sinensis* samples were grouped into a unique ITS sequence cluster under 95% similarity and two *O. sinensis* samples and six non-*O. sinensis* samples showed false claims. Our data showed that the ITS region could provide accurate species identification for *O. sinensis* samples, especially when macroscopic and microscopic method could not be applied in mixed commercial products.

Previous studies have compared the discriminatory power of different barcoding candidates in *O. sinensis* and its counterfeit identification (Zhang et al., 2009; Xiang et al., 2013). The probability of correct identification to species level of each barcoding amplicon wasn’t the same for different species (Xu, 2016). Most studies did the research through tree based method, such as maximum parsimony, neighbor-joining, and maximum likelihood analysis (Zhang et al., 2009; Quan et al., 2014). No study has compared the discriminatory power of different barcoding candidates in *O. sinensis* through a generally acknowledged index. Simpson’s index of diversity has been widely used for the typing method evaluation in molecular epidemiological research (Demczuk et al., 2017; Ramonaite et al., 2017). In this study, the ITS sequence showed the highest discrimination power among ITS, LSU and SSU region determined by Simpson’s index for 43 tested samples. The ITS sequence difference among samples from different species was significantly higher than those among samples from the same species. The length of ITS region is approximately 600 bp and consists of two variable spacers, ITS-1 and ITS-2, which are separated by the highly conserved 5.8S rRNA gene (Schoch et al., 2012). Although both ITS1 and ITS2 are the most common metagenomics sequencing amplicons in fungal community studies (Tonge et al., 2014; Motooka et al., 2017), our data showed ITS-2 had a higher discriminatory power than ITS-1 and should be the optimal metagenomics sequencing target for *O. sinensis* mixed samples in the future studies.

Since *P. hepiali* was recovered from the natural *O. sinensis* fruiting bodies as an endoparasitic fungus (Yang et al., 2008), it is also widely used in Asian countries for various potential pharmacological activities similar to *O. sinensis* (Thakur et al., 2011; Wang et al., 2016). In this study, 17 ITS clones from individual fruiting bodies were sequenced and no *P. hepiali* sequence was found which further confirmed that *P. hepiali* was not the dominant fungi in the natural *O. sinensis* fruiting bodies (Yang et al., 2008).

*Ophiocordyceps sinensis* parasitizes more than 50 different species of caterpillar larvae including *Hapialus* and *Thitarodes* distributed in the Tibetan Plateau (Wang and Yao, 2011). The morphology identification of *O. sinensis* fruiting bodies highly relies on experience and is hard to establish new species morphology recognition criteria (Xiang et al., 2013). Furthermore, it takes more than 10 years to train a traditional fungal taxonomist, the number of these experts decreased dramatically in the past decades. In recent years, ITS region has become the consensus primary fungal barcode (Schoch et al., 2012). Our data showed ITS could differentiate *O. sinensis* from other non-*O. sinensis* samples at cluster level of 95% similarity, much better than the other two barcoding candidates in this study because of the high similarity among different species. A unique *O. sinensis* cluster was identified from samples of different sources in this study. Even the closest ITS gene sequence cluster to *O. sinensis* which was *O. lanpingensis* showed higher than 7% variation.

In this study, we found two *O. sinensis* samples and six non-*O. sinensis* samples had false claims through ITS sequencing. *C. gunnii*, a synonym of *C. hawkesii* that parasitizes the larvae of *Napialus hunanensis*, is a natural counterfeit. Although the morphology is similar, the ITS sequence showed high variation that further confirmed the necessity to establish ITS identification criteria for *O. sinensis*. Besides, ITS sequence has multiple copies in a single cell which makes it possible to amplify this gene from tiny amounts of samples. Although different *O. sinensis* PCR identification methods have been developed (Peng et al., 2013; Hou et al., 2017), none of them have been accepted by the Chinese pharmacopeia.

## Conclusion

The ITS region showed the highest discrimination power as determined by Simpson’s index of diversity among 43 tested samples which could identify *O. sinensis* to species level. Since the authentication of *O. sinensis* related products is essential to ensure its safety and efficacy, *O. sinensis* identification either through ITS sequence variation comparison or unique PCR amplification of the species specific target, such as the ITS region, should be considered in the next revision of Chinese pharmacopeia, especially for those highly processed products.

## Author Contributions

SC designed this study. PZ and XR carried out the experimental work and data analysis. SK and FW collected and analyzed the samples. BL and SM guided the experiments.

## Conflict of Interest Statement

The authors declare that the research was conducted in the absence of any commercial or financial relationships that could be construed as a potential conflict of interest.

## Footnotes

1 http://www.sda.gov.cn/WS01/CL0847/146100.html

2 http://www.ncbi.nlm.nih.gov/BLAST/
